# Improving reproducibility in animal research

**DOI:** 10.1038/s41598-020-76398-3

**Published:** 2020-11-06

**Authors:** Florian Frommlet

**Affiliations:** Center for Medical Statistics, Informatics and Intelligent Systems, Section for Medical Statistics, Medical University Vienna, Vienna, Austria

**Keywords:** Preclinical research, Experimental models of disease

## Abstract

There have been increasingly lively discussions about many published scientific results failing validation by independent studies. This so-called reproducibility crisis has led to particularly strong criticism of methodological weaknesses in animal research. Inappropriate statistical methods, poor experimental design, and extreme standardization in trial design are some contributing factors to the problem. The purpose of this Collection is to present original methodologies to improve the status quo and additionally to report meta-research about the reproducibility of published animal research.

Within the last decade, it has become increasingly clear that many published scientific results, even those in prestigious journals, do not withstand the test of reproduction by independent research. Statisticians have been aware of the underlying problem for a long time, see for example Steven Goodman^1^. In his most famous paper^2^, John Ioannidis quite accurately predicted the frequency of false positive results one can expect in the scientific literature. Nevertheless, it came as quite a shock—to many researchers in different fields—when they had to admit a large proportion of their published findings were wrong. This culminated in the so-called reproducibility crisis.

Preclinical research has come under particular scrutiny because the stakes are so high, both in terms of research funding and societal impact^3^. Some of the problems involved are quite well known. For instance, poor statistical training of researchers within this area leads to poor experimental design, and the use of inappropriate statistical methods. More recently, researchers have become aware that extreme standardization in animal trials may also contribute to the problem^4^, with slight deviations in test conditions yielding different results. This Collection gathers together methods which aim to improve the status quo, and meta-research on the reproducibility of published animal research. The question as to the extent that the results from animal research can be translated into practical medicine is also touched on by some articles.

The group of John Ioannidis contributed a meta-analytic paper, for studies on domestic dogs with spontaneously occurring cancers. Such studies are of particular interest from a translational point of view, because dogs as model organisms are closer to humans than murine models. However, the results from Tan et al.^5^ clearly show that most of the studies performed in this area suffer severely from a lack of statistical power and proper reporting, both of methods and results.

Two contributions investigated the extent to which study designs that employ heterogenization can improve the reproducibility of animal trials. The group of Helene Richter shows that the simple step of performing experiments at different times of the day introduces sufficient systematic variation to obtain more replicable results^6^. Natasha Karp and her colleagues^7^ consider a multi-batch approach, where the study is planned for several small independent mini-experiments within the same lab. The idea is very similar to the approach to experimental replication that I have recently suggested myself^8^, except that in addition to studying the question of statistical analysis in this context, Karp et al.^7^ also demonstrate the actual performance in two case studies.

The group of Hanno Würbel presents some slightly contrasting results in their paper^9^. They systematically studied the influence of weaning time and housing conditions on a variety of behavioral and physiological outcome variables, where they observed smaller effects than expected. They conclude that more research is needed to understand which variables across labs are actually responsible for different outcomes in replication studies.

Stan Lazic contributes two papers, covering statistical aspects of the reproducibility problem. The first article^10^ presents a Bayesian alternative to the analysis of covariance, in the context of analyzing organ weight changes. The second article^11^ introduces a Bayesian prediction model to deal with pseudoreplications. Treating correlated data points as independent is among the most prevalent sources of statistical malpractice in animal trials. This should be rectified, either by using mixed models, or by a Bayesian approach as suggested here.

Two more contributions discuss cage effects. The first one^12^ studies cage-clustered-data, in the context of research on diets and gut microbiomes. The more theoretical paper by Michael Festing^13^ stresses the importance of taking into account cage effects at the level of study design. He gives the clear recommendation of using randomized block designs and corresponding methods for statistical analysis. However, a brief survey of pre-clinical studies shows that this important concept is currently hardly ever implemented in practice.

The Collection also includes papers directly concerned with the practical aspects of animal trials. One contribution, from Andelius et al.^14^, studies how insertion trauma of intracerebral devices affects variability of outcome measurements. They conclude that the time to stabilization differs depending on the device. This is important information for obtaining valid baseline values, and may have an effect on the reproducibility of studies using microdialysis. Another paper introduces PiDose, a system that automatically weighs the individual animals of a study, and based on that, provides the daily dosage of drug solution through each animal’s drinking water^15^. This system allows a reduction in interaction with experimenters, and thus, potentially helps decrease study bias. Finally, the Cardioprotection Large Animal Platform (CIBER-CLAP) is presented by Rossello et al.^16^, which was established in Spain to ascertain whether results from cardiovascular animal trials are reproducible, before the next step is taken, and greater complexity is introduced in a clinical setting.

Although the Collection has now been published, submissions are still welcome on a rolling basis. Papers accepted will then be added to the Collection as and when they are published. I would like to thank all the authors who have contributed to this Special Collection and I sincerely hope that their work presented here will help to improve the reproducibility of preclinical animal trials.
